# Patterns of Trauma Presentation in Ilorin, Nigeria: A 15-Year Review

**DOI:** 10.4314/ejhs.v35i1.6

**Published:** 2025-01

**Authors:** Gbadebo Hakeem Ibraheem, Abdur-Rasheed Adegoke Nasir, Olasunkanmi Misbaudeen Babalola, Lukman Olajide Abdur-Rahman, Babatunde Akibu Solagberu

**Affiliations:** 1 Centre for Injury Research and Safety Promotion, Department of Surgery, University of Ilorin Teaching Hospital, Ilorin, Nigeria; 2 Department of Surgery, Lagos State University, Ikeja, Nigeria

**Keywords:** Trauma, Injury, Road Traffic Crash, Pre-hospital Care, Emergency Medical Care

## Abstract

**Background:**

Injuries make up a significant portion of the emergency surgical patient load in most hospitals, representing a major public health threat The burden of injury as a public health issue is especially pronounced in low- and middle-income countries, where injuries are responsible for up to 90% of mortality. Identifying common mechanisms of trauma and patterns of presentation can aid in identifying at-risk populations and in the development of targeted preventive protocols.

**Methods:**

From February 2000 to January 2015, a prospective observational study was conducted to examine the patterns of presentation and epidemiology of patients presenting to the surgical emergency department of a University Teaching Hospital. All patients who presented during these 15 years were enrolled in the study.

**Results:**

A total of 27,588 patients were admitted through the surgical emergency department during the study period. Of these, 18,374 patients (66.6%) presented with trauma, while the remaining 33.4% sought care for other surgical emergencies. Trauma patients were generally younger, with an average age of 30.27 years, compared to an average age of 41.33 years for those presenting with other surgical emergencies. Road traffic crashes accounted for the majority of injuries (65.3%), followed by falls (8.9%) and assaults (6.9%). The majority of patients (69.2%) were transported to the hospital by friends and relatives.

**Conclusion:**

Injuries represent a substantial portion of the emergency surgical care demands in this setting. Road traffic injuries continue to be the predominant cause of trauma, with young adult males being the most frequent victims.

## Introduction

Trauma accounts for a significant portion of the emergency surgical patient load in most hospitals, posing a major public health threat. It is the leading cause of death among individuals aged 1 to 44 years worldwide, responsible for up to 72% of deaths in some age groups (e.g., 15–24 years) (1). The public health burden of injury is particularly severe in low- and middle-income countries (LMICs), where trauma accounts for up to 90% of mortality (2). In 2010, injuries caused 5.1 million deaths, surpassing the cumulative mortality from more prominent public health concerns in LMICs, such as HIV/AIDS, malaria, and tuberculosis, which together caused 3.8 million deaths (3).

Injuries arise from a variety of causes, which can be classified by mechanism (e.g., road traffic crashes, falls, violence), body part injured (e.g., head, limbs), or the nature of the causative factor (e.g., penetrating, blunt, barotrauma). More recently, the WHO's International Classification of External Causes of Injuries (ICECI) has been used for classification (4). Understanding injury mechanisms is essential for effective prevention strategies. According to the World Health Organization, annual road traffic deaths have reached 1.35 million, with road traffic crashes (RTCs) now being the leading cause of death among individuals aged 5–29 years (5). The burden of road traffic deaths and injuries (90%) disproportionately affects LMICs, a situation exacerbated by rapid urbanization and motorization (6).

While population health has benefited from increased focus on disease prevention, injuries often receive less attention than communicable and non-communicable diseases, despite their significant impact. Effective prevention and emergency care can reduce the burden of injuries. To develop targeted prevention strategies, it is crucial to understand the mechanisms and epidemiology of trauma. Identifying common trauma mechanisms and patterns can help pinpoint at-risk populations and inform the design of specific preventive measures. This study examines the epidemiology and patterns of trauma among patients presenting to the emergency department of a university teaching hospital in North-Central Nigeria over a 15-year period.

## Patients and Methods

Between February 2000 and January 2015, all patients who presented to the surgical emergency department of the University of Ilorin Teaching Hospital were enrolled in a prospective observational study to assess the epidemiology and patterns of trauma presentation. The hospital serves as the primary tertiary healthcare facility for Kwara State and a referral center for parts of five neighboring states in North-Central and South-West Nigeria. Data were collected using a pretested questionnaire completed by the admitting physician at the time of presentation. The questionnaire captured patients' demographic information, diagnosis, and, for trauma patients, the type of injury and source of transport to the hospital. Transport sources were categorized by who transported the patients: relatives and friends, bystanders, ambulances, or the police/Federal Road Safety Corps (FRSC). Data analysis was performed using IBM-SPSS version 24.

Ethical approval was obtained from the hospital's ethical review committee before the study commenced. The study was self-funded.

## Results

A total of 27,588 patients were admitted to the surgical emergency ward during the study period. Of these, 18,374 patients (66.6%) presented with trauma, while the remaining 33.4% presented with other surgical emergencies. The majority of trauma patients (72.4%) were male, with a male-to-female ratio of 2.2:1 among trauma victims and 1.55:1 among non-trauma surgical patients. The average age of trauma patients was 30.27 years, compared to 41.33 years for non-trauma patients. RTCs accounted for the majority of injuries (65.5%), followed by falls (8.9%) and assaults (6.9%). Occupational injuries and sports injuries were less common, contributing only 1.4% and 0.5% of cases, respectively. Road traffic crashes predominated across all age groups, though children had a relatively higher proportion of burns and falls compared to adults. Regarding transportation, 69.2% of patients were brought to the hospital by relatives or friends, while 12.7% were brought by the police or the FRSC.

## Discussion

Trauma has often been described as an “unrecognized epidemic” or a “malignant epidemic” to highlight its significant impact on public health (7, 8). At our hospital, trauma accounted for 66.6% of surgical emergency presentations, a finding consistent with studies in Pakistan and Kenya, where 71.8% and 73.5% of surgical emergencies were trauma-related, respectively (9, 10). This underscores the substantial burden trauma places on healthcare systems in LMICs.

The majority of trauma patients were young men, with an average age of 30 years, consistent with findings from other studies where young men are most affected by traumatic injuries (11, 12). This group represents the most active and economically productive segment of the population. Traumatic injuries in young men result in significant lifetime costs due to treatment expenses, lost income, productivity losses, and premature death.

Understanding the mechanisms of injury is crucial for guiding immediate patient management and predicting injury patterns. In our study, RTCs were the leading cause of injury (65.3%), followed by falls (8.9%) and assaults (6.9%). Road traffic crashes were the predominant cause of injury across all age groups, though children had a relatively higher incidence of burns and falls. These findings mirror those in other LMIC urban settings, where RTCs are the leading cause of injury, followed by falls and assaults, with children experiencing a higher proportion of burns and falls (10, 12, 13). The high rate of road traffic injuries is particularly concerning given the lack of effective pre-hospital care and emergency medical services in our region. In contrast, many higher-income countries have implemented strategies to reduce road traffic crashes, leading to shifts in injury mechanisms and patterns (14, 15). Improved driver education and better enforcement of traffic laws are potential strategies to mitigate RTCs in our community (16).

Most patients (69.2%) were transported to the hospital by relatives or friends, a rate higher than the 53% reported in previous studies from the same hospital (17). This highlights the importance of pre-hospital care, especially since many trauma victims are transported by untrained individuals. Only 12.7% of patients were transported by the police or the FRSC, underscoring the need to expand pre-hospital care training beyond law enforcement to the general public. Training laypersons in basic trauma care has been shown to improve survival rates in Cambodia and Iraq, suggesting this could be a viable strategy in our setting (18, 19).

While this study's data on specific injuries were incomplete and the time from injury to hospital arrival was not reliably recorded, the findings highlight the critical role of trauma in our population's health. Effective strategies for preventing RTCs and improving pre-hospital care are urgently needed. Investing in formal emergency medical services and expanding public education on trauma care could significantly reduce morbidity and mortality from injuries in LMICs.

## Figures and Tables

**Table 1:** Demographic distribution of patients presenting to the surgical emergency

		Trauma	Non-trauma	Total
**Gender**	Male	13,303	5,601	
	Female	5,071	3,613	
	Total	18,374	9,214	27,588
**Age distribution**	0 - 10 years	2,351	577	
	11 – 20 years	2,709	1,181	
	21 – 30 years	5,540	1,858	
	31 – 40 years	3,789	1,337	
	41 – 50 years	2,039	1,134	
	51 – 60 years	1,060	1,128	
	61 – 70 years	528	1,087	
	> 70 years	358	912	
	Total	18,374	9,214	27,588

**Table 2:** Demographic distribution of various mechanisms of injury

Mechanism	Sex distribution					Age distribution (year)					Total

	M	F	0-10	11-20	21-30	31-40	41-50	51-60	61-70	>70	
Road Traffic Injury	9033	3001	998	1626	3841	2774	1492	721	371	211	12034
Fall	1106	534	573	343	221	147	109	78	83	86	1640
Burns	451	266	271	142	131	88	44	20	13	8	717
Domestic	374	180	126	87	104	73	56	39	40	29	554
Assault	1020	247	41	246	512	278	102	53	21	14	1267
Gun shot injury	665	76	9	102	278	204	97	31	12	8	741
Occupational Injury	230	27	0	35	98	76	27	13	7	1	257
Sport	74	18	2	45	31	7	4	0	2	1	92
Others	750	322	83	151	244	160	132	127	108	67	1072
Total	13703	4671	2103	2777	5460	3807	2063	1082	657	425	18374

**Figure 1:**
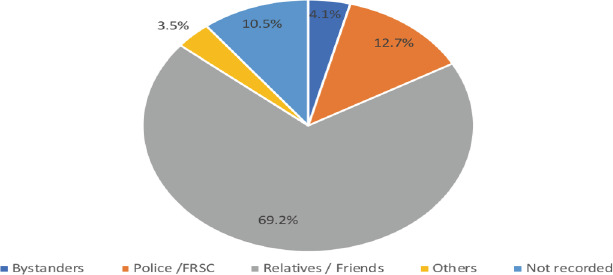
Pre-hospital transport
